# Recombination Difference between Sexes: A Role for Haploid Selection

**DOI:** 10.1371/journal.pbio.0030063

**Published:** 2005-02-22

**Authors:** Thomas Lenormand, Julien Dutheil

**Affiliations:** **1**UMR 5175Centre d'Ecologie Fonctionnelle et Evolutive, MontpellierFrance; **2**UMR 5171Université Montpellier II, MontpellierFrance; University of Bath

## Abstract

Why the autosomal recombination rate differs between female and male meiosis in most species has been a genetic enigma since the early study of meiosis. Some hypotheses have been put forward to explain this widespread phenomenon and, up to now, only one fact has emerged clearly: In species in which meiosis is achiasmate in one sex, it is the heterogametic one. This pattern, known as the Haldane-Huxley rule, is thought to be a side effect, on autosomes, of the suppression of recombination between the sex chromosomes. However, this rule does not hold for heterochiasmate species (i.e., species in which recombination is present in both sexes but varies quantitatively between sexes) and does not apply to species lacking sex chromosomes, such as hermaphroditic plants. In this paper, we show that in plants, heterochiasmy is due to a male-female difference in gametic selection and is not influenced by the presence of heteromorphic sex chromosomes. This finding provides strong empirical support in favour of a population genetic explanation for the evolution of heterochiasmy and, more broadly, for the evolution of sex and recombination.

## Abstract 2

Measuring the opportunity for natural selection on gametes provides the first empirical evidence for a theory explaining why recombination at meiosis varies between males and females

## Introduction

Sex differences in recombination were discovered in the first linkage studies on *Drosophila* [1,2] and *Bombyx* (Tanaka [1914] in [3]) almost one century ago. However, this observation remains today largely unexplained despite several attempts. Based on very limited observations (see Table 1), especially of *Bombyx,* in which the female is heterogametic, Haldane [3] suggested, as far as “these facts are anything more than a coincidence,” that the lower autosomal recombination rate in the heterogametic sex may reflect a pleiotropic consequence of selection against recombination between the sex chromosomes. Later, Huxley [4] showed that *Gammarus* males also recombined less than females. He gave the same evolutionary explanation, although he restricted it to cases of a marked sex difference.

**Table 1:**
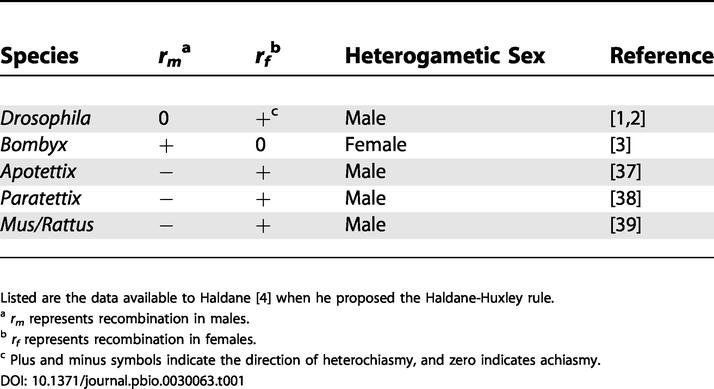
Data on Which the Haldane-Huxley Rule is Based

Listed are the data available to Haldane [4] when he proposed the Haldane-Huxley rule

^a^ *r_m_* represents recombination in males

^b^ *r_f_* represents recombination in females

^c^ Plus and minus symbols indicate the direction of heterochiasmy, and zero indicates achiasmy

This conjecture has now been confirmed for achiasmate species (i.e., species in which only one sex recombines) and is referred to as Haldane-Huxley rule: Nei [5] showed theoretically that tight linkage should evolve on Y or W chromosomes, and Bell [6] compiled a large dataset showing that achiasmy evolved 29–34 times independently, each time with no recombination in the heterogametic sex.

However, for heterochiasmate species, three problems with the Haldane-Huxley pleiotropy explanation were discovered [7,8]. The first problem arose when substantial variation in male-female differences in recombination rate was found between pairs of autosomes within mice [8] and *Tribolium* [9,10], and between genotypes for the same pair of autosomes [11]. The second problem was the discovery that hermaphrodite species (the platyhelminth *Dendrocoelum* [12] and the plant *Allium* [13]) may present strong heterochiasmy between male and female meiosis despite having no sex chromosomes or even sex-determining loci. The third problem was the discovery of species in which the heterogametic sex recombines more than the homogametic one (e.g., in some *Triturus* species) [14]. Because of these contradictory observations, variation in heterochiasmy has remained difficult to explain because of the absence of an alternative theory as well as the lack of a clear pattern in the data.

In 1969, Nei [5] worked out the first “modifier” model to study the evolution of sex differences in recombination, and concluded for autosomes that “the evolutionary mechanism of these sex differences is not known at present.” Surveying an updated dataset, Bell [6] concluded that “female gametes experience more crossing over among hermaphroditic plants (and perhaps animals), but this is not invariably the case among gonochoric animals (…) certainly (this) has never received any explanation.” The idea that heterochiasmy may be explained by a sex rather than by a sex chromosome effect, which was ignored by Haldane because of *Bombyx,* was reconsidered. This led Trivers [15] to suggest that, because only males with very good gene combinations reproduce (relative to females, for whom reproduction success is often less variable), they should recombine less to keep intact these combinations. He accounted for exceptions by variation in the regime of sexual selection. The idea was criticized by Burt et al. [16], who also questioned the correlations—with an updated dataset—between heterochiasmy and either sex or heterogamety. These authors tried to correlate the level of heterochiasmy with the amount of “opportunity for sex-specific selection,” but failed to find an effect. They were tempted to advocate neutrality, but were puzzled by the positive correlation between male and female recombination rate and by evidence showing compensation (e.g., female mice tend to recombine more on the X, as if they were compensating for no recombination in males; similarly, no species is known with achiasmy in both sexes [16]). In 1994, Korol et al. [17] insisted on a possible role for gametic selection but did not give evidence in favour of this claim. Recently, Lenormand [18], using Nei's modifier approach, showed that it is very difficult to explain heterochiasmy by sex-specific diploid selection. Rather, a sex difference in selection during haploid phase, or a sex difference in diploid selection on imprinted genes, is a more likely explanation. He predicted that, as far as haploid selection is concerned, the sex experiencing the more intense haploid selection should recombine less. Indeed, when allelic effects interact to determine fitness (i.e., when there is “epistasis,” either negative or positive), recombining decreases mean fitness in the population of the next generation [19]. This effect occurs because recombination breaks up combinations of genes that have previously been built up by selection. For a given average recombination rate between sexes and for a given average epistasis between male and female haploids, it is always advantageous for the haploid population (male or female) with the greatest absolute value in epistasis to be produced with the lowest amount of recombination. In this way, the “recombination load” that the haploid population is exposed to is minimized.

In this paper, we would like to come up with a more quantitative evaluation of the possible role of haploid selection in shaping heterochiasmy. For that purpose, we first updated the dataset of Burt et al. [16] on heterochiasmy, focusing on genetic maps that have become available over the last 15 years. We then determined how fast heterochiasmy evolves, in order to measure the amount of phylogenetic inertia on this trait. Finally, we determined whether variables such as gender, heterogamety, or the opportunity for selection in the haploid phase, could explain variation in heterochiasmy. If there is selection with substantial epistasis on some genes during the haploid phase, we expect the sex with the greater opportunity for haploid selection to show less recombination. Alternatively, if selection during the haploid phase is weak or without substantial epistasis, we do not expect it to produce a directional bias in the amount of recombination displayed by either sex.

## Results/Discussion

### Sex Chromosomes

Heterochiasmy is a fast-evolving trait, and phylogenetic inertia does not satisfactorily explain its distribution. In contrast to achiasmy, we found that heterochiasmy is not influenced by the nature of the sex chromosomes. This is interesting, because it suggests that achiasmy and heterochiasmy are influenced by qualitatively different evolutionary forces, although they seem to differ only quantitatively. It would be useful to determine whether achiasmy evolved to reduce the average recombination rate or to change the relative amount of recombination between the sexes. The two situations may be discriminated by determining whether the homogametic sex in achiasmate species tends to recombine more than in closely related chiasmate species. Evidence for such compensation would indicate that achiasmy did not evolve to reduce the average recombination rate. In the absence of such compensation, however, achiasmy may simply reflect selection for tight linkage. In such a situation, we propose that Haldane-Huxley rule may be caused by the converse argument to the one previously considered: The presence of achiasmy only in the heterogametic sex may reflect selection to maintain nonzero recombination rate on X or Z chromosomes in the homogametic sex. In species in which the average autosomal recombination rate is selected against (i.e., towards a lower equilibrium value), loss-of-function (recombination) mutations with an effect restricted to one sex may spread only if they affect the heterogametic sex, because mutations suppressing recombination in the homogametic sex completely suppress recombination on the X or Z chromosome. The same argument applies to XO species and may explain why achiasmy is associated only with the heterogametic sex. In addition, this hypothesis does not require the existence of genes suppressing recombination between the sex chromosomes with autosomal pleiotropic effects. Under this hypothesis, there is no reason to find an effect of the presence of heteromorphic sex chromosome on the amount of heterochiasmy, as originally envisioned by Haldane and Huxley. Overall, this hypothesis would explain why heterochiasmy and achiasmy differ qualitatively and why we do not observe any effect of sex chromosomes on heterochiasmy.

### Heterochiasmy in Animals

In animals, male-female dimorphism in haploid selection may also contribute to heterochiasmy. In general, there is no female haploid phase in animals, because meiosis is completed only at fertilisation. As far as at least some genes are expressed and under selection during the male haploid phase, this would tend to bias towards tighter linkage in males. Sets of genes responsible for male-specific meiotic drive systems would be good candidates and are often found in tight linkage. Measuring the opportunity for haploid selection in animals may be possible within some groups. Imprinting may, however, act as a confounding effect in many groups of animals while trying to measure the opportunity for “haploid” selection. Within-species comparisons of imprinted regions or of regions with sex-specific recombination using high-resolution maps [20] may be more fruitful to discriminate among potential causes of heterochiasmy in animals. In particular, there is evidence in humans that the reduction in crossing-over associated with imprinting is in the direction that theory predicts, even if this pattern is consistent with other explanations [21]. Finally, understanding exceptions within groups (e.g., male marsupials, contrarily to most mammals, recombine more than females of the species [22]) may also shed light on the different hypotheses.

### Heterochiasmy in Plants

We found that plant heterochiasmy is correlated with the opportunity for male and female haploid selection. Female meiosis tends to exhibit lower recombination rates relative to male meiosis when selection is intense among female gametophytes (e.g., in Pinaceae) or mild among male gametophytes (e.g., in highly selfing species). This pattern is expected if heterochiasmy is determined by the relative magnitude of haploid selection in male and female individuals. Finding a pattern consistent with this general population genetic prediction is, of course, not firm evidence that male-female dimorphism in haploid selection is the evolutionary force generating heterochiasmy. Other correlates of selfing rates might have to be closely examined [23]. However, we consider this explanation the most parsimonious so far. Our finding provides, therefore, the first empirical evidence for a theory explaining male-female differences in the amount of recombination and contributes to our understanding of contradictory observations that have puzzled geneticists for almost a century. It also indicates that the amount of recombination may be shaped by indirect selection, and, therefore, corroborates theories based on selection and variation for the evolution of sexual reproduction.

## Materials and Methods

### 

#### An extended dataset

We measured heterochiasmy as the log of the male/-to-female ratio (*ρ*) of autosomal recombination rate measured either with chiasma number or map length. We log-transformed the ratio to avoid bias due to measurement error in the denominator. Chiasma-count data for different species were compiled by Burt et al. [16], and we used their dataset, adding a few recent studies. We compiled genetic map data and linkage studies in animals and plants for which both a male and a female map were available. Only homologous fragments (i.e., between shared markers) in male and female maps were considered (especially in low-resolution maps). Heterochiasmy data were available for 107 species, with 46 sets of data based on genomic maps (Table 2).

**Table 2:**
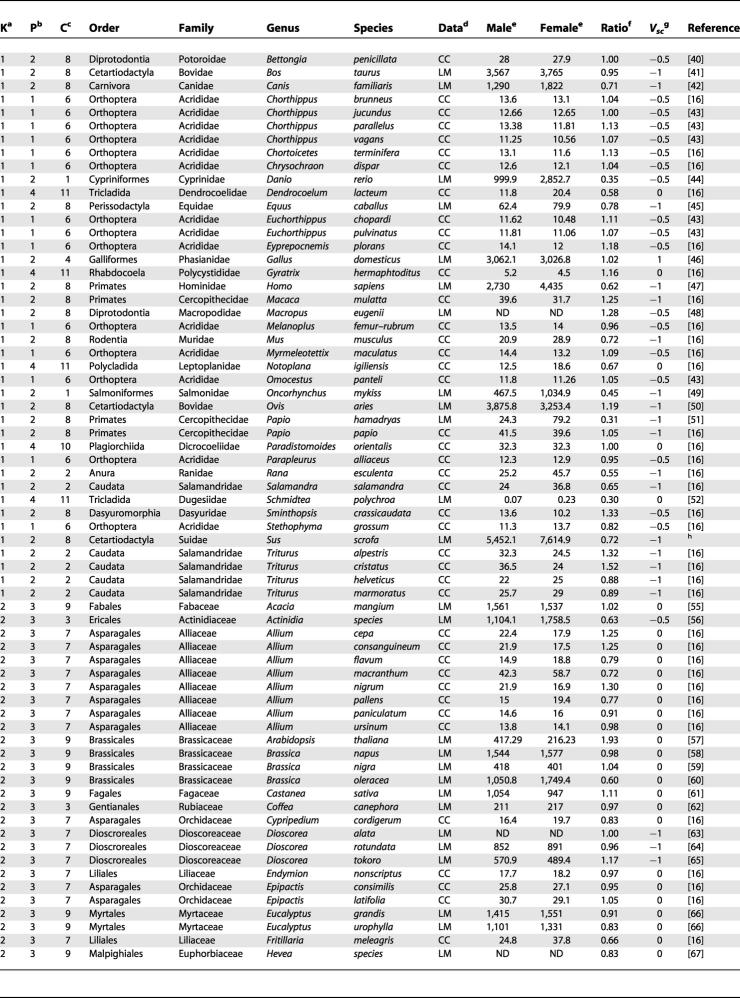
Dataset Pooled by Species with Levels of Phylogenetic Grouping Used in the Analysis

Note that references given in Burt et al. [17] were not repeated here

^a^ K, kingdom. Numeric indicators in this column are: 1, Animalia; 2, Plantae

^b^ P, phylum. Numeric indicators in this column are: 1, Arthropoda; 2, Chordata; 3, Embryophyta; 4, Platyhelminthes

^c^ C, class. Numeric indicators in this column are: 1, Actinopterygii; 2, Amphibia; 3, Magnoliopsidae (subclass asterids); 4, Aves; 5, Coniferopsida; 6, Insecta; 7, Liliopsida; 8, Mammalia; 9, Magnoliopsidae (subclass rosids); 10, Trematoda; 11, Turbellaria

^d^ Data refers to linkage map (LM) or chiasma count (CC)

^e^ Male and female indicate the value for the chiasma count or map length for each sex

^f^ Ratio refers to male/female recombination rate

^g^ *V_sc_* refers to the presence or absence of sex chromosome (see Materials and Methods, “Sex chromosome effect”)

^h^ Data were obtained from maps DBNordic2 and NIAIJapan (http://www.genome.iastate.edu/pig.html) [54,55]

ND, no data

**Table 2:**
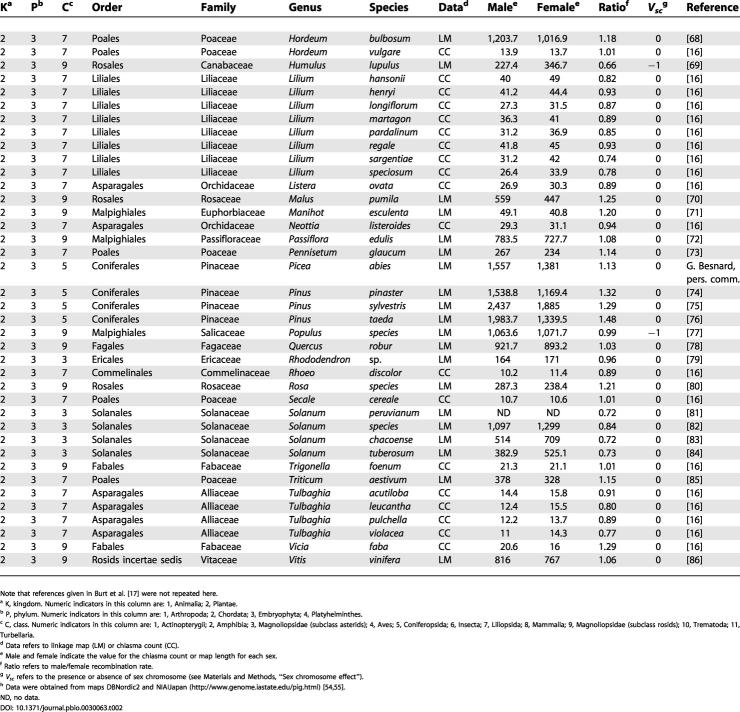
Continued

#### Phylogenetic inertia

Heterochiasmy may evolve so slowly that there is important phylogenetic inertia. Alternatively, it may be so fast-evolving that the amount of heterochiasmy takes on nearly independent values among related species. In the same way, heterochiasmy may be so variable between genotypes within a species that it may be difficult to measure and irrelevant to analyse species specific effects. In order to get a picture of phylogenetic inertia on heterochiasmy, we estimated the phylogenetic autocorrelation of *ρ* using Moran's *I* spatial autocorrelation statistic [24]. When standardized, values of Moran's *I* vary from −1 to 1. Positive values indicate that heterochiasmy is more similar than random within a taxonomic level, whereas negative values indicate that it is more different. Because a few species had multiple estimates of heterochiasmy, we also estimated the within-species correlation. The resulting correlogram is shown in Figure 1. We found that heterochiasmy is a fast-evolving trait: Genotypes tend to be correlated within a species (I/I_max_ = 0.38, *p* = 7.9%), but this correlation is lower among species within genera (I/I_max_ = 0.18, P-value = 13%), and very low when comparing genera within families (I/I_max_ = 0.039, *p* = 63%). This pattern is very different from the one observed for highly autocorrelated traits using the same method (for instance, mammalian body size [25]). This analysis indicates that there is very little phylogenetic inertia overall on heterochiasmy, but that the species level is appropriate for our dataset. However, this low level of inertia may nevertheless inflate type-I error while testing the effect of independent variables on heterochiasmy. In order to avoid this problem, we tested the association between different variables and heterochiasmy using a generalized estimating equations linear model correcting for the full phylogeny (see below) [26].

**Figure 1:**
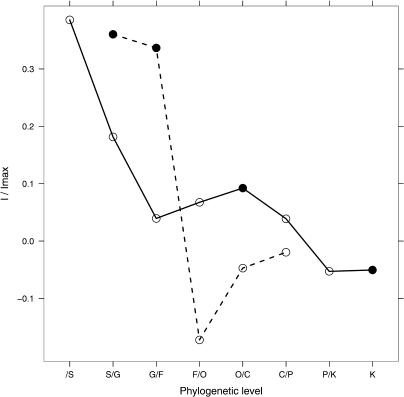
Phylogenetic Correlogram of Heterochiasmy and Selfing Rate The y-axis represents Moran's *I* rescaled to enable comparisons between each taxonomic level for heterochiasmy (*ρ*, solid line) and selfing rate (*V_m_*, dashed line). The x-axis represents the taxonomic level: /S is the correlation within species, S/G is the correlation of species within genera, etc. F, family; O, order; C, class; P, phylum; K, kingdom. Filled points indicate significance at *p* = 0.05.

#### Sex chromosome effect

For each species, we reported the presence of sex chromosomes. We defined the variable *V_sc_* with the following values: −1 for XY/XX species, −1/2 for XO/XX or XY/XX without pseudoautosomal regions (marsupials), 0 for species without sex-chromosomes, and +1 for ZZ/ZW species. We distinguished the −1 and −1/2 cases to reflect the fact that, in the latter, recombination does not occur between sex chromosomes, so we expect a lower current selection pressure to suppress recombination. Under the Haldane-Huxley hypothesis, the presence of sex chromosomes is supposed to favour reduced recombination rate in the heterogametic sex. We therefore expect a positive effect of the variable *V_sc_* on *ρ*. We did not find such an effect in animals or plants (the linear effect of *V_sc_* on *ρ* is not significantly different from zero [*p* = 0.75 in animals and *p* = 0.52 in plants], assuming species were independent), and this result is unchanged if the −1 and −1/2 cases are not distinguished. Given this negative result, there was no need to do a phylogenetic correction.

#### Gametic selection

In animals from our dataset, there is no female haploid phase because the completion of meiosis occurs only at fertilisation (sperm triggers the end of meiosis). In male gametes, very few genes are expressed, and sperm phenotype is determined mostly either by the diploid genotype of the paternal tissue or by its mitochondrial genome. Imprinted genes, which can also affect the evolution of heterochiasmy [18,21], may be as numerous as haploid-expressed genes and act as a confounding factor while evaluating the “opportunity” for male or female gametic selection. As a consequence, we did not attempt to evaluate the opportunity for haploid selection in animals. Rather, we focused on plants, in which there is both a male (pollen) and female (ovule) haploid phase and during which many genes are expressed (e.g., as many as 60% of genes may be expressed in the male gametophyte [27,28]).

In order to evaluate the effect of the “opportunity for selection” for male haploid phase on *ρ,* we used selfing rate as an indirect variable estimating the degree of pollen competition. We assume that with high selfing rates, there is less genetic variation among competing pollen grains and, therefore, less scope for haploid selection. We defined *V_m_* (the degree of male gamete competition in plants) using three values depending on the amount of selfing: 0 for dioecious, self-incompatible or largely outcrossing (less than 5% selfing reported) species; 1 for species exhibiting low selfing rates (less than 30% reported); and 2 for other species. We used these three broad categories to reflect the fact that selfing rate is often variable within species and that it is often measured indirectly and with low precision. We therefore expect a positive effect of the variable *V_m_* on *ρ* if the opportunity for male gametic selection favours smaller *ρ* values, as predicted by the modifier model [18]. We tested this effect using the 57 species for which we were able to estimate *V_m_* (Table 3). We used a linear model in R [29] assuming that all species are either independent or phylogenetically related. In the latter case, we used a generalized estimating equations linear model [26] with a plant phylogenetic tree to the family level using data from Davies et al. [30], and several calibration points, including the *Picea*/*Pinus* divergence approximately 140 million years ago [31], that are not included in the Davies et al. dataset. We found an effect in the right direction with or without correcting for the phylogeny (linear effect of *ρ* on *V_m_*, *p* < 0.0002 in both cases, Figure 2). The fact that selfing plants exhibit higher recombination rates than their outcrossing relatives has been mentioned previously in the literature [32,33]. However, in most cases, recombination was measured only in male meiosis. It would be valuable to reexamine this trend in the light of our results that recombination in male meiosis is typically greater than in female meiosis among selfers.

**Figure 2:**
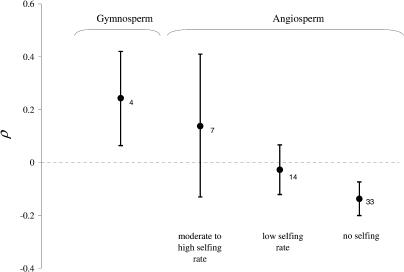
Logarithm of Male-Female Ratio in Recombination Rate in Plants Mean and 95% confidence interval of *ρ* is shown for different groups of plants, assuming normality and independent data points The number of species in each group is indicated next to the mean.

**Table 3:**
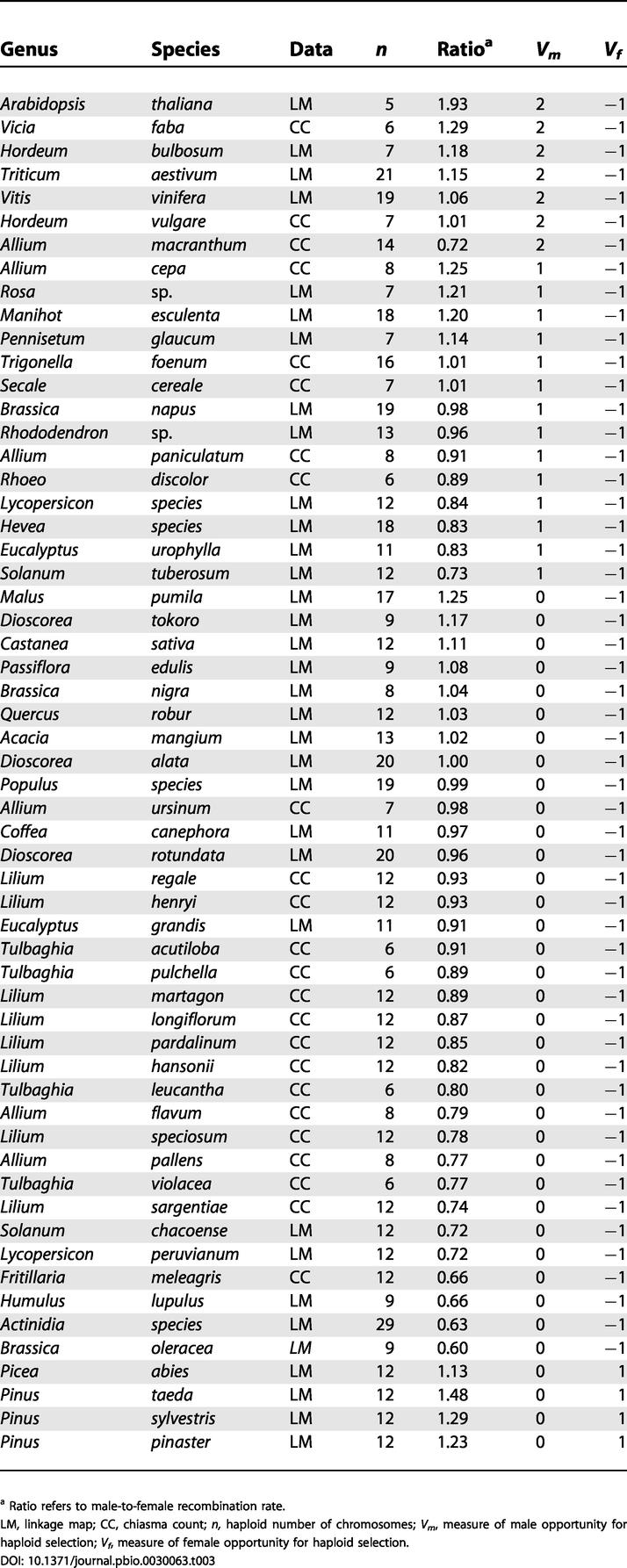
Plant Species Used to Test the Effect of Male and Female Opportunity for Selection

^a^ Ratio refers to male-to-female recombination rate

LM, linkage map; CC, chiasma count; *n,* haploid number of chromosomes; *V_m_*, measure of male opportunity for haploid selection; *V_f_*, measure of female opportunity for haploid selection

In order to evaluate the effect of the “opportunity for selection” during the female haploid phase on *ρ* in plants, we contrasted angiosperms with gymnosperms. In angiosperms, ovules do not compete much with each other on a mother plant, because resource accumulation starts after fertilisation (i.e., during fruit development in the diploid phase). In *Pinus* (three species in our dataset; see Table 2), male meiosis, female meiosis, and pollination occur in the year prior to fertilisation, but the pollen tube stops growing until the next spring, while the female gametophytes continue to accumulate resources and compete with each other over the course of the year. The same situation occurs in *Picea,* although the period between female meiosis and fertilisation is only 2–3 mo [34]. Perhaps more importantly, the endosperm (which is the organ managing resources for the zygote) is haploid in Pinaceae, in contrast to the double fertilisation that occurs in angiosperms to produce at least a diploid (typically triploid) endosperm [35,36]. We therefore expect that *ρ* should be greater in Pinaceae, compared to angiosperms. We assigned *V_f_* (the degree of female gamete competition in plants) the values 1 for gymnosperms and −1 for angiosperms. We expected a positive effect of the variable *V_f_* on *ρ* according to the modifier model. An effect in the right direction was indeed detected (linear effect of *V_f_* on *ρ, p* = 0.011 and *p* = 0.0001, with and without correcting for the phylogeny as above, respectively; see Figure 2).
